# Supplementary material for: Strategies to maintain recovery from alcohol problems during the COVID-19 pandemic: Insights from a mixed-methods national survey of adults in the United States
Source: PLoS One. 2023 Apr 17;18(4):e0284435. doi: 10.1371/journal.pone.0284435 (PMC10109499; doi:10.1371/journal.pone.0284435)
Supplement: S1 Appendix — (DOCX) [file pone.0284435.s001.docx]

| **Theme** | **Code**  **(label)** | **Definition**  **(what it is)** | **Boundary**  **(what it is not)** | **Examples**  **(illustrative quotes)** |
| --- | --- | --- | --- | --- |
| Active Pursuits | Hobbies, recreation & creative activity | Mentions of hobbies (either broadly such as “hobbies” or specifically such as painting, reading, video games, etc. | Not physical activities or exercise, working/employment, or being in nature/outdoors | “Worked more on hobbies. Doing business with my hobby”  “Video games”  “Immersion in games with family”  “Leer libros” [reading books]  “I started taking piano lessons” |
|  | Keeping busy | Explicit mentions of “keeping busy,” “staying busy” or staying productive | Not specific activities captured under other codes, e.g., biking (physical activity & health), work (work), doing work on the house (home enhancement), being in nature (nature & outdoors)  Not mentions of seeking normalcy or keeping a routine only | “Keeping my body and mind busy”  “Keeping occupied”  “Staying productive and busy”  “Estar ocupado haciendo algo alrededor de la casa” [being busy doing something around the house] |
|  | Work | Mentions of working/employment being helpful in maintaining recovery | Not “working on a project” or “working around the house”  Not mentions of seeking normalcy or keeping a routine only | “Working at home.”  “Staying busy with work.”  “estar enfocado en mi trabajo” [being focused on my job] |
|  | Maintaining normalcy and a schedule | Making a conscious effort to continue lives as normally as possible, not changing much. Mentions of keeping a routine or structure  Emphasizes normalcy and routine | Not keeping busy (although statements may be double coded with it) | “Continuing to live my life as normally as possible”  “Keeping to a routine”  “Commitment to my regime”  “keeping things the same” |
|  | Home enhancement | Mentions of projects around the house, yard work, work on the house, planting, painting house, etc. | Not activities that simply happen in or around the house, in which home improvement is not the focus | “Doing fixing up the things around the house, such as painting ,yardwork and fixing the fence”  “yard work”  “doing home repairs and completing them” |
| Spirituality & Meditation | Religion, spirituality & faith | Personal relationships with the church, a religion, or engaging in faith practices, such as praying, worshiping  or reading from scriptures that help maintain recovery. | Any discussion of mindfulness, meditation, or positive thinking. | “Prayer, remembering god and keeping my trust in him strong!”    “Read the Bible, pray more.”    “Spiritual relationship with God” |
|  | Mindfulness or meditation | Mentions of meditation as a method (either alone or listed alongside other activities) to maintain recovery | Anything that does not mention meditation or mindfulness specifically (i.e., “praying/ connecting with a higher power”) or that has been listed alongside meditation many times but unless meditation is specifically mentioned we cannot count this | “Daily spiritual meditation and communicating with others in recovery.”    “During COVID-19, I have found meditation to be helpful, as well as nature walks.”    “Meditation apps on cell phone.”    “Mindfulness and using the time to catch up on things I wanted to do.”    “ Living a mindful life.” |
| Using Lay Support | Mutual-help groups | Any mention of AA or 12-step groups, including jargon (e.g., Big Book, higher power) or slogans (e.g., one day at a time). Can include in person sessions, online, or outside of sessions | Family, friends, partners, or any licensed professional supporting them during this time. | “Daily reflections from AA”    “AA meetings, ours were small and never stopped during COVID.”  “Modifying my recovery program to respect social distancing protocols by meeting online.” |
|  | Peers in recovery | Any mention of peers/friends/others who are in recovery or in mutual-help groups/AA | Mentions of friends, family, or social support generally (not specifically those in recovery) | “Staying connected with AA friends.”  “Talking to people in AA.”  “Talking on the phone with my AA sponsor, and also with my husband who is also in recovery.” |
| Confidence in Recovery | Long-term or established recovery | Statements indicating that the participant feels secure in their recovery, there has been a long amount of time since experiencing problems, or considering oneself recovered | Thinking about the consequences or previous reflections pertaining to negative experiences caused by drinking. | “My recovery was over 30 years ago, so it isn’t an issue at all anymore.”    “I have been sober for 34 years and I prefer to live rather than pick up a drink and destroy what I have worked so hard to achieve.”    “I have not the least desire to ever drink alcohol since I totally quit in 1987” |
| Cognitive Strategies | Willpower | Descriptions or mentions of willpower, determination, commitment, confidence in ability to maintain recovery; can include actions such as “just saying no”; also can include mentions of “me/myself/the person in the mirror”, etc. | *Any*action to help maintain sobriety. Not desiring to drink anymore. | “Will power and commitment.”    “Belief in GOD! Very strong willpower.”    “Sticking to my personal commitment to never drink again, resolve I suppose.”    “Support of wife, and my determination to not drink beyond my abilities to safely drink.”    “The person in the MIRROR.”    “Just saying NO!!!” |
|  | Acknowledgement of future problems | Acknowledgement/  anticipation of the consequences of drinking on oneself or one’s family or peers, or the acknowledgment of one’s own limits with regards to drinking, as a cautionary method to help maintain recovery | Only the admission of a drinking problem | “Knowing my limits.”  “Knowing that I don’t want to drink again and seeing how it as affected my friends who continue to drink.”  “Knowing that things will only get worse if I drink.”  “I am afraid of what will happen if I drink.”  “Both of my parents were alcoholics. I have seen the underbelly of the animal and chosen not to participate.” |
|  | Recollections of past problems | Any thought or reflection of personal past negative experiences with alcohol that help them limit or abstain from drinking excessively now.    Recalling a negative consequence associated with drinking that led them to recovery. | Abstaining or reduced drinking due to long periods of time.  Negative experiences that other people in their lives had with alcohol (we can usually classify this under “problem recognition” instead)    A health problem or condition that caused them to reduce their drinking. | “All I have to do is weigh the difference between life before alcohol abuse and after alcohol abuse: 180-degree improvement. Going back would be stupid.”    “Remembering how bad rock bottom was and never wanting to go back there.”    “Reminding myself how I don’t want to go back to who I was when I drank nor experience how I felt when I drank. I like feeling like a human now.” |
|  | Staying positive or practicing gratitude | Any mention of staying positive, positive thinking, or practicing gratitude as a method to help maintain recovery. | Does not include mentions of mindfulness or remaining calm/keeping a calm mind. | “Positivismo.”  “Keeping a positive mindset about the accomplishments I have made to improving my life.”  “Positive thinking”  “Gratitude and meditation. Just happy to be here! […] I wake up and name 3 things I am grateful for!” |
|  | Cognitive or emotional regulation | Responses highlighting thought processes and/or the use of intentional strategies to modify thinking or to manage emotional reactions. | Not statements that could be captured under Mindfulness & Meditation or Religion & Spirituality. | “Being self aware and noticing triggers.”    “Keeping a clear mind, and thinking before acting on anything.”  “Mind over matter” |
| Staying Connected | Family | Highlighting family members as supportive factors re abstaining/limiting intake of alcohol/maintaining recovery  Mentions of family members health or wellbeing as a focus during COVID-19  Can include family members in recovery | Remembering a family member who was harmed by drinking   Drinking alongside  family members    Recalling an ultimatum set by a family member as a reason to stop drinking in the past | “A pact I made with my brother that both of us would no longer use alcohol.”    “Family time”; “my love for my family.”    “Being there for my children.”    “My daughters wellbeing.”    “Helping my wife who has cancer. She is more important.” |
|  | Friends | Highlighting friends/  contact with friends as supportive factors re abstaining/limiting  intake of alcohol/maintaining recovery  Can also be used in responses expressing that friends have been unhelpful in recovery (i.e., how not being around friends actually makes it easier not to drink) | Remembering a friend who was harmed by drinking   Recalling an ultimatum set by a friend as a reason to stop drinking in the past  A more general mention of social support or support systems without the explicit mention of friends  Specifying friends in recovery, this would be under “peers in recovery” | “Contact with friends with texting, e mail or phone.”  “Reading, calling family zoom celebrations for friends & families birthdays and exercise.”  “Only had 2 beers with friends no friends come by now because on virus.”  “Staying away from liquor stores and old friends.” |
|  | Social support | Mentions of general social support or support systems without explicit mention of friends/family/  significant others, etc.  Can include either receiving social support or providing social support | Explicit mentions of friends/family/  significant others, etc.  Mentions of support systems specific to recovery, ie. Recovery community, others in recovery, sponsors/sponsees | “I have a strong support system.”  “My support network and my faith.”  “Helping others.” |
| Unexpected Deterrents | COVID restrictions | Mentions of COVID-19-specific restrictions (ie. venues closing, social distancing, quarantining, etc.) or consequences related to restrictions (ie. not feeling comfortable going out even if bars are open) making it easier to not engage in excessive/any alcohol use (and maintain recovery) by removing opportunities or temptations to drink  Can include restrictions that prevent other people from drinking, and as a result making it easier for the speaker not to drink | Restrictions unrelated to or not resulting from COVID-19-specific  prevention measures | “Bars not open.”    “Inaccessibility to social drinking has curbed lots of relapses.”    “COVID-19 has actually helped keep me from dealing with social situations where I would be more likely to want to drink.”    “Nothing--actually it's been easier without seeing people having fun at parties at the restaurant I work at.”    “No social situations of others drinking due to quarantines.”    “Just the isolation and the fact that I am uncomfortable going out to a bar to meet friends due to the COVID.” |
|  | Health issues | Mentions of current **or** past physical or mental health conditions (whether they did or did not occur as a result of drinking) that deter/have deterred people from excessive/any alcohol use  Worry about contracting a health condition in the future from drinking, that deters people from excessive/any alcohol use and helps them maintain recovery | Coping by helping someone else with a health condition  (i.e “helping my wife who has cancer. She is more important.”)    Refusing to drink because of someone else with a health condition (i.e., “One of my best friends was an alcoholic and passed away in her early thirties. Seeing her struggle with her alcoholism had an impact on me well before she passed and I strived to never drink around her and limit my intake.”) | “I had cancer 5 years ago and due to long term effects from treatments I no longer drink alcohol.”    “Bladder issues do not allow me to consume more than one drink of alcohol.”    “Last time I drank I tried to commit suicide and I will never go back there.”    “I have no desire to drink and 2 years ago I had a laryngectomy which made the taste of alcohol abhorrent.”    “I decided a few years ago that my heart health is more important than anything that damages it.”    “I have been experiencing lots of physical pain since the beginning of 2020, which has mostly killed my desire to drink. Since I feel pretty bad on a daily basis, I don’t want to add the discomfort of a hangover or the pain of acid reflux caused by binge.” |
| Ensuring Wellness | Physical activity & wellness | Mentions of physical exercise or other physical activities (walking, biking, etc.) that have been helpful in maintaining recovery  Can also be taken as “staying active” or mentions of sports  Mentions of staying healthy, taking care of mental/physical health, eating right, etc. | Activities that do not explicitly state that physical activity is happening  ex. “being outdoors” are not to be counted as exercise; however, “staying healthy” can count as wellness even if physical activity was not specified | “Walking and exercise.”  “Staying positive and active.”  “Getting out of the house and doing something physical, like walking, hiking, swimming.”  “Trying to remain physically healthy. I have diabetes and drinking makes things worse for me, especially during this pandemic.”  “Exercise and keeping healthy habits.”  “Taking care to eat right, exercise”. |
|  | Healthcare | Includes prescription medication, therapy or counseling, providers such as therapists or doctors, healthcare clinics or programs that have been helpful in maintaining recovery | Not AA, recovery residences, or treatment considered not “medical” | “My family and recovery program (outpatient counseling).”  “Help from a therapist, enrolling in Medicaid, support from family and friends, finding work again.” |
|  | Nature & outdoors | Mentions of being in nature or outdoors as a way that has been helpful in maintaining recovery | Working or being in the yard | “Enjoying nature in my backyard.”  “Talking about it with my spouse, acknowledging it and more time in nature or doing meaningful activities.”  “Being outdoors.” |
| Substance Use | Controlling exposure to substances | Descriptions of individual behaviors that help keep an individual safe while drinking such.  Individuals being proactive with their decisions to remain “safe” while drinking.  Can also include intentional actions to avoid drinking. | Does not include thinking of negative consequences associated with drinking, or health issues that discourage drinking. | “Not keeping alcohol in the house.”  “Continue to limit my drinking to occasional activities with friends once a month or less and no more than 1 or 2 drinks.”  “I don’t drink because I haven’t done anything that deserves a reward. And when I do drink I only have two drinks. Then I stop.”  “I do not drink during the work week.” |
|  | Using substances to cope | Mentions of drugs or substances in general (can be cannabis or other recreational drugs) that have been helpful in maintaining recovery | Using alcohol  Using prescription medication | “Bud/cannabis/  weed/marijuana/  pot”  “Using drugs instead.” |
| Alternative Strategies | Alternative strategies | Strategies that do not fall into other categories that have been helpful in maintaining recovery  Includes “not thinking about it” and “ignoring it” | Strategies that do fall into other categories listed above | “Spending time away from work.”  “Being home and spending time with myself.”  “My dogs.”  “Sleeping.”  “Not thinking about it/ignoring it” |
