# Supplementary material for: Strategies to maintain recovery from alcohol problems during the COVID-19 pandemic: Insights from a mixed-methods national survey of adults in the United States
Source: PLoS One. 2023 Apr 17;18(4):e0284435. doi: 10.1371/journal.pone.0284435 (PMC10109499; doi:10.1371/journal.pone.0284435)
Supplement: Attachment — Submitted filename: Response to Reviewers.docx [file pone.0284435.s002.docx]

**REVIEWER #1**

**Thank you for an excellent and clear presentation of this wonderfully readable piece of work. My only main feedback would be if you would rework the abstract ever so slightly to more clearly indicate where the sample was drawn from. It becomes very clear in the text but the abstract does leave one wondering.**

Thank you for the compliment on this paper and the suggestion to improve it. We have added further details about the source of our sample to the Abstract.

**The other feedback I might add is if you could say very very briefly why you went with the survey tools you chose. It need not be long, something to the effect of "it has been used frequently in this population or similar populations and is reliable etc etc". I would argue that the findings here are actually not applicable to just dramatic events but to recovery literature more broadly.**

We have added a statement about our selection of the coping responses items from the Environmental Influences on Child Health Outcomes survey to the Materials and Methods section.

**REVIEWER #2**

**Thank you for the opportunity to review this very interesting and relevant paper. This is very well written paper that will be of interest to the readership of the journal. My comments/suggestions are minor and are related to some inconsistencies or need for clarification.**

Thank you for the compliments and the suggestions to strengthen the paper that follow.

**Abstract. Line 36. You mentioned 10 themes but didn't list all 10 in the abstract. Also, in the main text there are only 7 themes and in the figure 2, 9 themes. Please make sure you correct these sections so there is consistency. 10 themes is a lot and seems that some of the 9 themes in figure 2 are very small and perhaps there is some overlap so they can be merged?**

We apologize for the confusing presentation. In the original version, we described only the top seven themes out of a concern for manuscript length. We have taken advantage of this revision to include descriptions all ten thematic clusters. Although the reviewer suggests that ten are a lot, we find that they are substantively distinct. Thus, we present all ten themes. In addition, we have removed the count of themes from the Abstract to avoid potential confusion and confirmed that Figure 1 (previously mislabeled in the original submission) shows the ten thematic clusters.

**Methods. Line 148, I presume this is ethical approval? if so, please be explicit.**

We have expanded the statement in question to state that we received ethics board approval by the university’s institutional review board.

**Line 163. If lifetime AUD symptoms were collected before the survey, should come before describing the survey.**

Lifetime AUD symptoms were collected as part of the survey. The AUDIT -Cquestions were part of the eligibility screening and were used to identify and exclude potential participants who reported current heavy drinking, which we presumed would be incompatible with being in recovery.

**Line 166. Was this classification arbitrary or based on previous scales?**

Our classification of AUD severity followed the DSM-5 diagnostic criteria. We have revised the line in question to make this clear.

**Line 188. better to say in statistical package/software R, otherwise confusing for those that are not familiar with it. Also for consistency with saying dedoose software.**

We have rephrased the statement so that it is clear that R is a statistical software package.

**Line 193. Did you use thematic analysis? Braun and Clarke?**

Yes, we conducted a thematic analysis of open-ended responses following procedures described by Saldana in The Coding Manual for Qualitative Researchers.

**Results: Table 1: bracket missing at very good or good QoL and recovery length over 5 years.**

We have corrected the table at the places indicated.

**Themes: in main text 7 themes, in figure 2, 9 themes. please be consistent and delete remove/merge as necessary.**

As noted above, we have taken advantage of this opportunity to describe all ten themes that were identified. We have reviewed the manuscript to ensure consistency.

**ADDITIONAL EDITOR COMMENTS**

**Please include evidence of IRB approval and/or a statement of ethical clearance.**

We have expanded the statement about receiving institutional board approval in the Materials and Methods section.

**Also please clarify your sample population and the source population from which the sample was drawn.**

We have revised the Abstract based on Reviewer #1’s suggestion to make the sample source clearer and have added a reference for additional information about it in the Materials and Methods section.

**The number of themes should be consistent in the narratives and tables throughout the manuscript.**

We believe the changes made in response to Review #2’s comments have addressed this concern.
